# Transparency-optimized position–position control for human-scale bilateral teleoperation

**DOI:** 10.3389/frobt.2026.1797952

**Published:** 2026-05-29

**Authors:** Amir Noohian, Faezeh Haghverd, Alan Lynch, Martin Jagersand

**Affiliations:** 1 Department of Electrical and Computer Engineering, University of Alberta, Edmonton, AB, Canada; 2 Department of Computing Science, University of Alberta, Edmonton, AB, Canada

**Keywords:** bilateral teleoperation, dynamic compensation, human-scale teleoperation system, inverse dynamics, position–position control

## Abstract

The position–position (P–P) architecture is a simple and inherently stable control strategy for bilateral teleoperation systems, but its transparency is fundamentally limited by robot dynamics, particularly in human-scale teleoperation, where large inertia and damping degrade operator perception in free motion. In this paper, we propose a transparency-optimized P–P control architecture that leverages estimated inverse dynamics to compensate for the leader and follower robot dynamics without relying on force/torque sensors. The proposed controller preserves the simplicity and stability of the P–P scheme while significantly improving transparency. The approach is implemented and experimentally validated on a human-scale WAM bilateral teleoperation system. Experimental results show that the proposed architecture substantially improves free-motion transparency, reducing joint position tracking error by up to 32% and leader-side impedance by up to 56% compared to a gravity-compensated P–P controller. In hard-contact scenarios, the proposed method maintains comparable maximum transmittable impedance while achieving up to 53% improvement in force tracking accuracy. These results demonstrate that dynamic compensation can significantly enhance the practical transparency of P–P teleoperation systems for human-scale robots without increasing hardware complexity.

## 1 Introduction

Bilateral teleoperation systems are a cornerstone of human–machine interaction, enabling operators to perform complex manipulation tasks in remote or hazardous environments with increased precision and safety. Such systems are widely used in applications including telesurgery (Maeso et al., 2010; Smits et al., 2025), space exploration (Fong et al., 2013), search and rescue, surveillance (Khasawneh et al., 2019), and explosive ordnance disposal (Zhou et al., 2024). In these scenarios, the operator commands a remote robot (follower) through a local interface (leader), with the goal of executing tasks accurately while safely interacting with the environment. Effective teleoperation relies on the operator’s ability to perceive position, contact, and interaction forces in a coherent and intuitive manner, enabling precise and reliable task execution (Laghi et al., 2020).

Haptic interfaces used in teleoperation systems range from lightweight devices such as the Phantom Omni to human-scale systems like the DLR teleoperation facility HUG (Hulin et al., 2011). Compact devices offer ease of deployment and low inertia, but are often limited in workspace, force output, and realism of interaction. In contrast, human-scale teleoperation systems provide larger workspaces, higher force capabilities, and improved dexterity, allowing operators to perform tasks that closely resemble natural human motion. These advantages make human-scale systems particularly suitable for demanding manipulation tasks and high-fidelity interaction with remote environments. However, the increased mass and complexity of such systems also introduce significant control challenges, especially in achieving high-quality force feedback and responsive behavior.

A central performance objective in bilateral teleoperation is transparency, which refers to the degree to which the operator perceives the remote environment without distortion introduced by the teleoperation system. In the ideal case, the leader–follower pair and communication channels behave as a massless and infinitely stiff connection between the operator and the environment. Achieving such transparency requires accurate transmission of motion and interaction forces, as well as effective compensation of the robots’ inherent dynamics (Lawrence, 1993). Classical four-channel teleoperation architectures achieve this by exchanging both position and force information between the leader and follower robots. However, in practice, these architectures often rely on force/torque sensors, which increase system cost and complexity, are sensitive to noise, and can introduce stability and robustness issues—particularly in human-scale teleoperation systems.

To avoid these challenges, simpler control architectures have been widely adopted, among which the two-channel position–position (P–P) control scheme is one of the most common (Lawrence, 1993). The P–P architecture synchronizes the motion of the leader and follower robots using position feedback alone, while interaction forces are indirectly reflected to the operator through position tracking errors caused by contact between the follower robot and the environment. Due to its simplicity, inherent stability properties, and sensorless implementation, the P–P controller has been successfully applied in a variety of teleoperation systems (Aliaga et al., 2004). Nevertheless, the basic P–P architecture exhibits fundamental limitations in transparency performance, particularly with respect to force reflection and impedance matching (Lawrence, 1993; Ni and Wang, 2004).

A key challenge in the P–P architecture arises from conflicting transparency requirements in free-motion and contact conditions. During free motion, high transparency requires accurate position tracking at the follower side and low apparent impedance at the leader side, enabling smooth and responsive operator motion. This typically necessitates high follower gains and low leader gains. In contrast, during interaction with a stiff environment, equal control gains on both sides are required to ensure symmetric force reflection and stable contact behavior. These conflicting requirements prevent a fixed-gain P–P controller from achieving optimal transparency across different interaction regimes, resulting in reduced overall system performance.

**FIGURE 1:**
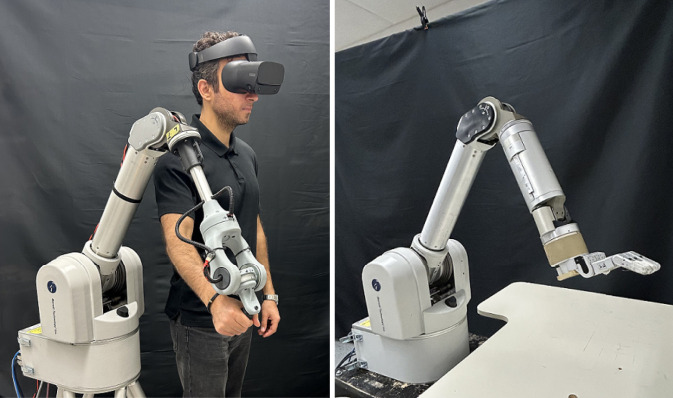
WAM bilateral teleoperation system setup with a 4-DOF leader arm equipped with a custom haptic wrist (left) and a 7-DOF follower arm (right). Figure contains images of the author(s) only.

To address these limitations, this paper proposes a transparency-optimized P-P control architecture based on inverse dynamics modeling of the leader and follower robots. The proposed approach incorporates dynamics-based feedforward compensation using estimated robot models, without relying on force/torque sensors or additional hardware. By compensating for the inherent dynamics of the teleoperation system, the proposed architecture expands the achievable transparency range of the P–P controller while preserving its simplicity and stability advantages. The effectiveness of the approach is validated experimentally on a customized bilateral WAM teleoperation system (Figure 1).

The main contributions of this work are:

- Characterization of the achievable transparency range of the P–P architecture when robot dynamics information is available.
- Estimation of the inverse dynamics of the leader and follower robots in a bilateral teleoperation system.
- Development of a transparency-enhanced P–P control architecture using dynamics-based feedforward compensation.
- Experimental implementation and transparency evaluation on a customized WAM bilateral teleoperation system.

## 2 Related works

### 2.1 Inverse dynamics estimation

Accurate inverse dynamics models are essential for implementing model-based control architectures in robotic systems. A widely adopted approach for obtaining inverse dynamics is robot dynamic parameter estimation, in which a set of dynamics parameters is identified using analytical (Gautier and Khalil, 1990) or numerical (Gautier, 1991) techniques. This methodology has been successfully applied to a variety of robotic manipulators, including KUKA LBR Med (Shao and Ficuciello, 2023), KUKA LBR iiwa (Stürz et al., 2017), Barrett WAM (Sousa and Cortesão, 2013), and Franka Emika Panda (Gaz et al., 2019). Previous studies have focused on improving estimation accuracy and reliability, for example, by enforcing physical feasibility of the identified parameters (Sousa and Cortesão, 2013; Stürz et al., 2017; Gaz et al., 2019) and by accounting for the mounting configuration of the manipulator (Tröbinger et al., 2023).

Inverse dynamics can also be obtained using learning-based methods, which typically require little or no prior knowledge of the robot model. These approaches include non-parametric techniques such as locally weighted projection regression (LWPR), Gaussian process regression (GPR) (Nguyen-Tuong et al., 2008), and local Gaussian process regression (LGP) (Nguyen-Tuong et al., 2009), as well as parametric methods based on artificial neural networks (ANN) (Morse et al., 2020; Hitzler et al., 2019). More recent work has incorporated physical priors into learning frameworks, including rigid-body kernels in GPR (Nguyen-Tuong and Peters, 2010), deep Lagrangian networks (DeLaN) (Lutter et al., 2019), and physics-informed neural networks (PINN) (Yang et al., 2023).

Compared to learning-based approaches, dynamic parameter estimation leverages explicit robot models and requires a significantly smaller number of parameters to represent the inverse dynamics. As a result, it offers improved robustness, interpretability, and generalization when deployed on real robotic hardware. These properties are particularly important in bilateral teleoperation systems, where model inaccuracies directly affect stability and transparency. For these reasons, this work adopts a parameter estimation approach to obtain the inverse dynamics of both the leader and follower robots in the WAM teleoperation system.

### 2.2 Transparency optimization in teleoperation systems

While inverse dynamics estimation has been extensively studied, its use for explicitly improving transparency in haptic teleoperation—particularly through real-world experimental validation—remains limited. One of the earliest and most influential works, Lawrence (1993), analyzed bilateral teleoperation systems from a dynamic perspective and demonstrated that achieving high transparency requires dynamic compensation in the position control loop of the four-channel architecture. In that study, however, the position–position (P–P) architecture was evaluated only in its basic form, without dynamic compensation, resulting in substantially reduced transparency compared to four-channel schemes.

Subsequent research explored the use of inverse dynamics and related techniques to enhance teleoperation performance. For example, Tufail (2015) applied inverse dynamics estimation within an impedance control framework for haptic teleoperation in homecare robotics. Adaptive control approaches based on inverse dynamics were proposed in Liu and Tavakoli (2011) to address nonlinearities in bilateral teleoperation systems, while Mohammadi et al. (2017) employed nonlinear disturbance observers to improve robustness, stability, and transparency. Despite promising results, these methods were predominantly validated in simulation or limited experimental setups, restricting their demonstrated applicability to practical, multi-degree-of-freedom teleoperation systems.

In the context of telesurgery, learning-based methods have been used to identify inverse dynamics and estimate external forces on the da Vinci Research Kit (Yilmaz et al., 2020). This work was later extended in Yilmaz et al. (2023), where a sensorless four-channel teleoperation architecture combined inverse dynamics compensation, external force estimation, and disturbance observers. While this approach achieved superior position and force tracking compared to two-channel architectures, it did not evaluate leader-side impedance, which is a critical metric for assessing the operator’s perceived transparency and ease of use.

In human-scale teleoperation, transparency is further challenged by the high inertia and damping of longer manipulators. Previous works have explored improving transparency through force feedforward (Fahmi and Hulin, 2018) and local force feedback (Hastrudi-Zaad and Salcudean, 1999; Balachandran et al., 2020). However, these approaches typically rely on end-effector force/torque sensors to measure interaction forces. While such sensors provide accurate measurements at the robot tip, they are costly and can only detect forces applied at the end-effector. This limitation becomes particularly significant in large robotic systems, where interactions often occur along the robot body due to contacts with the environment or unintended collisions. In such cases, body contacts remain undetected by end-effector sensors, which can limit both safety and transparency in human-scale teleoperation.

In this work, we focus on transparency optimization for the P–P teleoperation architecture through dynamics-based compensation and real-world experimental validation. Rather than relying solely on tracking performance, we evaluate transparency using operator-centered metrics, including leader-side impedance, which directly reflects the operator’s interaction experience. By emphasizing physical experiments and perceptual transparency measures, this study addresses a gap in the existing literature and provides practical insight into improving transparency in real bilateral teleoperation systems.

## 3 Methodology

### 3.1 General transparency analysis framework

A bilateral teleoperation system can be modeled using a two-port network representation. For a linearized one-degree-of-freedom (1-DOF) teleoperation system in the frequency domain, the leader and follower dynamics can be expressed as shown in Equation 1

$$\begin{array}{cc} Z_{l}X_{l} & =F_{h}+F_{l},\\ Z_{f}X_{f} & =-F_{e}+F_{f}, \end{array}$$ (1)

where $Z_{l}$ and $Z_{f}$ denote the linearized impedances of the leader and follower robots, $X_{l}$ and $X_{f}$ are the leader and follower positions, $F_{h}$ and $F_{e}$ represent the human and environment interaction forces, and $F_{l}$ and $F_{f}$ are the control efforts applied to the leader and follower robots, respectively. Throughout the paper, the subscripts l and f consistently refer to the leader and follower robots.

The transparency of the teleoperation system can be quantified using the hybrid matrix H , which relates the human force and follower position to the leader position and environment force as shown in Equation 2

$$\left[\begin{array}{c} F_{h}\\ X_{f} \end{array}\right]=\underset{H}{\underbrace{\left[\begin{array}{cc} h_{11} & h_{12}\\ h_{21} & h_{22} \end{array}\right]}}\left[\begin{array}{c} X_{l}\\ F_{e} \end{array}\right].$$ (2)

The hybrid parameters are defined in Equation 3 as

$$\begin{array}{cc} h_{11} & =\frac{F_{h}}{X_{l}}{|}_{F_{e}=0},\;h_{12}=\frac{F_{h}}{F_{e}}{|}_{X_{l}=0},\\ h_{21} & =\frac{X_{f}}{X_{l}}{|}_{F_{e}=0},\;h_{22}=\frac{X_{f}}{F_{e}}{|}_{X_{l}=0}. \end{array}$$ (3)

For ideal transparency, the operator should experience the remote environment as if directly connected to it, meaning identical motion on both sides $\left(X_{l}=X_{f}\right)$ should result in identical interaction forces $\left(F_{h}=F_{e}\right)$ (Lawrence, 1993). This condition can be equivalently expressed by requiring the transmitted impedance felt by the operator, defined in Equation 4, as

$$Z_{to}=\frac{F_{h}}{X_{l}},$$ (4)

to match the environment impedance, defined in Equation 5, as

$$Z_{e}=\frac{F_{e}}{X_{f}}.$$ (5)

Using the hybrid representation, the transmitted impedance can be written as a function of the environment impedance, as shown in Equation 6

$$Z_{to}=\frac{h_{11}+Z_{e}\left(h_{12}h_{21}-h_{11}h_{22}\right)}{1-h_{22}Z_{e}}.$$ (6)

According to Hannaford (1989), the necessary and sufficient condition for full transparency is that the hybrid matrix takes the form shown in Equation 7

$$H=\left[\begin{array}{cc} 0 & 1\\ 1 & 0 \end{array}\right],$$ (7)

which guarantees perfect position tracking and exact force reflection.

In practice, transparency is evaluated using parameters that are physically meaningful and experimentally measurable. In free motion, $h_{11}$ represents the apparent impedance of the leader robot as perceived by the operator, while $h_{21}$ quantifies the position tracking performance between the leader and follower robots. However, the parameters $h_{12}$ and $h_{22}$ correspond to scenarios in which the leader is rigidly constrained, which does not occur in typical teleoperation operation.

Following Aliaga et al. (2004), hard-contact behavior is instead characterized using the parameters defined in Equation 8

$$F_{12}=\frac{F_{h}}{F_{e}}{|}_{X_{f}=0},\;Z_{11}=\frac{F_{h}}{X_{l}}{|}_{X_{f}=0},$$ (8)

where $F_{12}$ measures force reflection accuracy during contact and $Z_{11}$ denotes the maximum transmittable impedance. Together, the parameters $h_{11}$ , $h_{21}$ , $F_{12}$ , and $Z_{11}$ provide a comprehensive and experimentally accessible characterization of teleoperation transparency across both free-motion and hard-contact conditions.

### 3.2 Position–position control architecture

In the position–position (P–P) control architecture, only the positions of the leader and follower robots are exchanged. The leader motion serves as the reference trajectory for the follower robot, while the follower position is simultaneously fed back as the reference for the leader. This symmetric structure enables bilateral coordination without explicit force sensing. Force reflection emerges implicitly when the controller reacts to position tracking errors caused by interactions between the follower robot and the environment.

**FIGURE 2:**
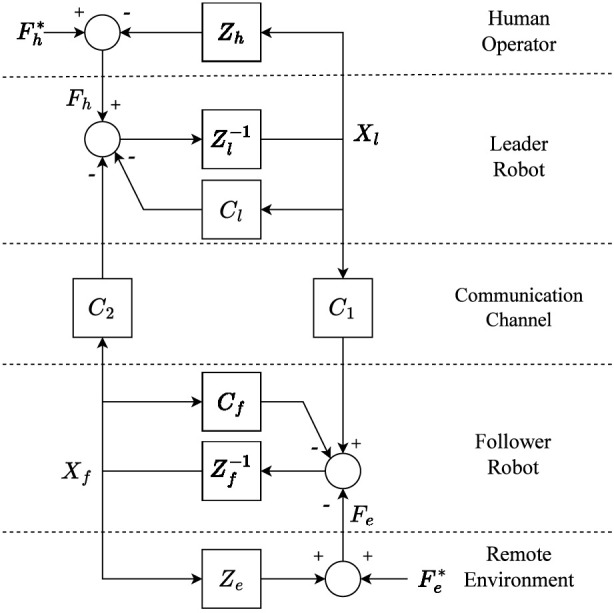
Position–position control architecture block diagram.

The controller efforts applied to the leader and follower robots can be written as shown in Equation 9

$$\begin{array}{cc} F_{l} & =-C_{2}X_{f}-C_{l}X_{l},\\ F_{f} & =C_{1}X_{l}-C_{f}X_{f}, \end{array}$$ (9)

where $C_{1}$ and $C_{2}$ are the position transmission controllers (see Figure 2). In the standard P–P architecture, these controllers are chosen as in Equation 10

$$C_{1}=C_{f},\;C_{2}=-C_{l},$$ (10)

where $C_{f}=k_{p}^{f}+k_{d}^{f}s$ and $C_{l}=k_{p}^{l}+k_{d}^{l}s$ are PD controllers for the follower and leader robots, respectively. Substituting Equation 10 into Equation 9, the control efforts reduce to Equation 11

$$\begin{array}{cc} F_{l}\left(s\right) & =C_{l}\left(X_{f}-X_{l}\right),\\ F_{f}\left(s\right) & =C_{f}\left(X_{l}-X_{f}\right). \end{array}$$ (11)

Using the general transparency framework described in Section 3.1, the transparency parameters of the P–P architecture can be derived as given in Equation 12

$$\begin{array}{cc} h_{11} & =Z_{l}+\frac{C_{l}Z_{f}}{Z_{f}+C_{f}},\\ h_{21} & =\frac{C_{f}}{Z_{f}+C_{f}},\\ F_{12} & =\frac{Z_{l}+C_{l}}{C_{f}},\\ Z_{11} & =Z_{l}+C_{l}. \end{array}$$ (12)

In free motion, high transparency requires a small apparent impedance at the leader side, meaning that $h_{11}$ should ideally be close to zero. In practice, this is not achievable due to the presence of the leader dynamics $Z_{l}$ , follower dynamics $Z_{f}$ , and the local controllers $C_{l}$ and $C_{f}$ . The leader impedance $Z_{l}$ can be interpreted as the *intervening impedance* introduced in Yokokohji and Yoshikawa (1994), which is transmitted to the operator as additional damping and centering stiffness to prevent drift (Ni and Wang, 2004). Consequently, the effective lower bound on the leader-side impedance in free motion becomes $h_{11}\approx Z_{l}$ . To approach this condition, the follower controller gains must be chosen significantly higher than the leader gains, such that $C_{f}\gg C_{l}$ , which improves position tracking while minimizing resistance felt by the operator. Although $h_{21}$ approaches unity when follower dynamics are neglected, in practice, finite bandwidth and unmodeled dynamics degrade position tracking performance, especially during fast operator motions.

In hard-contact conditions, transparency is primarily determined by force reflection. From Eqaution 12, accurate force tracking requires identical controller gains on the leader and follower sides. Furthermore, the expression for $Z_{11}$ indicates that when the follower is constrained by a stiff environment, the operator perceives only the dynamics of the leader robot and its local controller. As a result, sufficiently high leader gains are required for the operator to clearly feel rigid contacts.

In summary, transparency in the P–P architecture involves conflicting requirements across interaction modes. During free motion, transparency demands accurate position tracking at the follower side and low apparent impedance at the leader side, which is achieved using high follower gains and low leader gains. Conversely, during hard contact, high leader gains are necessary to convey contact stiffness, while ideal force reflection requires matched gains on both sides. These incompatible requirements lead to inherent trade-offs: optimizing transparency in one regime degrades performance in the other. Gain-switching strategies (Ni and Wang, 2004) can partially mitigate this issue but introduce additional stability concerns, and neither impedance shaping nor position tracking can be simultaneously optimized across all interaction conditions.

### 3.3 Transparency-optimized P–P control architecture

To address the conflicting transparency requirements of the basic P–P architecture, we propose incorporating model-based dynamic compensation directly into the position–position control loop. By leveraging the full inverse dynamics of both the leader and follower robots, the proposed controller compensates for the intrinsic robot dynamics in a feedforward manner. We demonstrate both theoretically and experimentally that this approach overcomes the fundamental transparency limitations of the basic P–P scheme while preserving its simplicity, sensorless nature, and stability properties.

To incorporate the leader and follower impedances into the P–P architecture, the position transmission channels are modified according to Equation 13

$$\begin{array}{cc} C_{1} & =Z_{f}+C_{f},\\ C_{2} & =-\left(Z_{l}+C_{l}\right), \end{array}$$ (13)

where $Z_{l}$ and $Z_{f}$ denote the estimated impedances of the leader and follower robots, and $C_{l}$ and $C_{f}$ are local PD controllers.

Using the modified communication channels in Eqaution 13, the resulting controller efforts can be written as Equation 14

$$\begin{array}{cc} F_{l}\left(s\right) & =Z_{l}X_{f}+C_{l}\left(X_{f}-X_{l}\right),\\ F_{f}\left(s\right) & =Z_{f}X_{l}+C_{f}\left(X_{l}-X_{f}\right). \end{array}$$ (14)

Each control input consists of two components: a feedforward term that compensates for the robot dynamics and a feedback term that enforces position synchronization.

Substituting Eqaution 14 into the general transparency analysis framework, the transparency parameters of the proposed controller are obtained in Eqaution 15

$$\begin{array}{cc} h_{11} & =0,\\ h_{21} & =1,\\ F_{12} & =\frac{Z_{l}+C_{l}}{Z_{f}+C_{f}},\\ Z_{11} & =Z_{l}+C_{l}. \end{array}$$ (15)

The values of $h_{11}=0$ and $h_{21}=1$ in Eqaution 15 indicate that, under accurate dynamic modeling, ideal transparency in free motion is achieved. Specifically, the operator experiences zero apparent impedance and perfect position tracking, matching the transparency conditions of the four-channel architecture.

In hard-contact conditions, force reflection is characterized by $F_{12}$ . For identical leader and follower robots with matched controller gains, ideal force tracking $\left(F_{12}=1\right)$ can be achieved. However, the maximum transmittable impedance $Z_{11}$ remains limited by the dynamics of the leader robot and its local position controller, as in the basic P–P architecture. Consequently, although interaction forces can be accurately reflected to the operator, the perceived stiffness of a rigid environment is bounded by the leader-side dynamics and control gains.

This limitation arises from the motion-based nature of position-controlled bilateral teleoperation, where force reflection fundamentally depends on position errors rather than direct force feedback. As a result, while the proposed controller eliminates transparency trade-offs in free motion, the infinite stiffness associated with ideal hard contact cannot be rendered without increasing leader-side gains.

In practice, the proposed feedforward dynamic compensation significantly improves transparency without introducing conflicting control requirements. In free motion, the system achieves full transparency comparable to the four-channel architecture without additional tuning constraints. In hard contact, effective force reflection is maintained through matched controller gains, while the maximum reflected stiffness remains bounded as in conventional P–P control. Compared to the four-channel gold standard, the only remaining compromise is reduced reflected stiffness. In return, the proposed approach provides a sensorless, cost-effective, stable, and straightforward solution for achieving high transparency in real-world teleoperation systems.

### 3.4 Inverse dynamics modeling for manipulators

A robot manipulator is considered as an open kinematic chain of n+1 rigid bodies and n rigid joints. The generalized coordinates $q\in R^{n}$ represent the configuration of the n joint angles. The dynamic model of the robot is given by Equation 16

$$M\;\left(q\right)\ddot{q}+b\;\left(q,\dot{q}\right)\dot{q}+g\;\left(q\right)+\tau_{d}=\tau ,$$ (16)

where $M\left(q\right)\in R^{n\times n}$ denotes the inertia matrix, $b\left(q,\dot{q}\right)\dot{q}\in R^{n}$ the centrifugal and Coriolis torque, $g\left(q\right)\in R^{n}$ the gravity torque, and $\tau \in R^{n}$ the actuation torque. Moreover, $\tau_{d}\in R^{n}$ represents the dissipative torque, mostly due to joint friction. For friction modeling, we evaluated several approaches (Desai and Howe, 2001; Colomé et al., 2015; Huang et al., 2025), but none yielded satisfactory performance in compensating the robot dynamics. The main difficulty was accurately capturing static and Coulomb friction, and the resulting models did not work well in practice, often leading to instability and loss of passivity. Therefore, only viscous friction was retained, leading to the friction model $\tau_{d}=F_{v}\dot{q}$ , where $F_{v}\in R^{n\times n}$ denotes the diagonal matrix of the viscous friction coefficients. The dynamic model in Eqaution 16 can be linearized with respect to a set of inertial parameters as given in Equation 17

$$\underset{\tau }{\underbrace{\left[\begin{array}{c} \tau_{1}\\ \tau_{2}\\ \vdots \\ \tau_{n} \end{array}\right]}}=\underset{Y\left(q,\dot{q},\ddot{q}\right)}{\underbrace{\left[\begin{array}{cccc} y_{11}^{⊤} & y_{12}^{⊤} & \cdots & y_{1n}^{⊤}\\ 0 & y_{22}^{⊤} & \cdots & y_{2n}^{⊤}\\ \vdots & \vdots & \ddots & \vdots \\ 0 & 0 & \cdots & y_{nn}^{⊤} \end{array}\right]}}\underset{\pi }{\underbrace{\left[\begin{array}{c} \pi_{1}\\ \pi_{2}\\ \vdots \\ \pi_{n} \end{array}\right]}},$$ (17)

where $Y\left(q,\dot{q},\ddot{q}\right)\in R^{n\times L}=\frac{\partial \tau }{\partial \pi }$ is called the model regressor. The computation of Y requires the joint position q , joint velocity $\dot{q}$ , and joint acceleration $\ddot{q}$ . The inertial parameters for link i is expressed in Equation 18 as

$$\begin{array}{c} \pi_{i}=\left[L_{xx,i},L_{xy,i},L_{xz,i},L_{yy,i},L_{yz,i},L_{zz,i},\right.\\ {\left.l_{x,i},l_{y,i},l_{x,i},m_{i},F_{vi}\right]}^{⊤}, \end{array}$$ (18)

where $L_{xx,i},L_{xy,i},L_{xz,i},L_{yy,i},L_{yz,i},L_{zz,i}$ are the components of the symmetric inertia matrix expressed in joint frame i , $m_{i}$ is the mass, and $f_{v,i}$ is the viscous friction coefficient. $l_{x,i},l_{y,i},l_{z,i}$ are the components of the first moments of mass through Equation 19

$${\left[l_{x,i},l_{y,i},l_{z,i}\right]}^{⊤}=m_{i}r_{c,i},$$ (19)

where $r_{c,i}\in R^{3}$ is the center of mass relative to the link frame i . Only a subset of the standard inertial parameters is necessary for modeling the robot dynamics. Moreover, some of those are not separately identifiable by regression methods. To obtain this set of parameters, a process of regrouping and combining the identifiable standard parameters is required. Either analytical (Gautier and Khalil, 1990) or numerical (Gautier, 1991) methods can be used. This minimum parameter set is called the robot’s base inertial parameters (Gautier and Khalil, 1990). The dynamics of the robotic manipulator can then be written as given in Equation 20

$$\tau =Y_{b}\left(q,\dot{q},\ddot{q}\right)\pi_{b},$$ (20)

where $Y_{b}\left(q,\dot{q},\ddot{q}\right)$ is the regressor matrix for the base parameters, and $\pi_{b}\in R^{b}$ is the base parameter vector. Having an excitation trajectory with N joint positions, velocities, accelerations, and torques, an over-determined linear system (N>b) with $\bar{\tau }\in R^{n.N}$ and ${\bar{Y}}_{b}\in R^{n.N\times b}$ is derived from Eqaution 20 as given in Eqaution 21

$$\bar{\tau }={\bar{Y}}_{b}\pi_{b}.$$ (21)

A least-square estimate of $\pi_{b}$ can be calculated (Gautier and Khalil, 1990; Gautier, 1991) using Equation 22

$${\hat{\pi }}_{b}={\left({\bar{Y}}_{b}^{⊤}{\bar{Y}}_{b}\right)}^{-1}{\bar{Y}}_{b}^{⊤}\bar{\tau }.$$ (22)

The excitation trajectory for each joint should be properly designed to excite the dynamic parameters and avoid the rank deficiency of the regressor matrix ${\bar{Y}}_{b}$ . The condition number of the regressor matrix is used to optimize the parameters of joint trajectories, which are modeled as finite Fourier series. For joint i , the trajectory $q_{i}\left(t\right)$ is expressed in Equation 23 as

$$q_{i}\left(t\right)=q_{i0}+\overset{M}{\underset{k=1}{\sum }}\left(a_{i,k}\sin\left(k\omega_{f}t\right)+b_{i,k}\cos\left(k\omega_{f}t\right)\right),$$ (23)

where $q_{i0}$ denotes the offset of the position trajectory, and $\omega_{f}$ is the fundamental angular frequency of the Fourier series with period $T_{f}=2\pi /\omega_{f}$ . The amplitudes of the Fourier series $a_{i,k}$ and $b_{i,k}$ are obtained so that they minimize the condition number of the regressor matrix.

### 3.5 External torque estimation

The dynamic model of a robot manipulator in Eqaution 16, in the case of the robot having contact with the environment, can be modified according to Equation 24 as

$$M\left(q\right)\ddot{q}+b\left(q,\dot{q}\right)\dot{q}+g\left(q\right)+\tau_{d}+\tau_{\mathrm{ext}}=\tau ,$$ (24)

where $\tau_{\mathrm{ext}}$ shows the external torque applied to the robot joints. Replacing the dynamic of the robot with the estimated dynamic from Eqaution 22, we have Eqaution 25

$$Y_{b}\left(q,\dot{q},\ddot{q}\right){\hat{\pi }}_{b}+\tau_{\mathrm{ext}}=\tau .$$ (25)

From Eqaution 25, the external torque can be estimated as follows in Eqaution 26

$${\hat{\tau }}_{\mathrm{ext}}=\tau -Y_{b}\left(q,\dot{q},\ddot{q}\right){\hat{\pi }}_{b}.$$ (26)

The external Cartesian wrench is related to the external joint torques through the manipulator Jacobian as given in Equation 27

$$\tau_{\mathrm{ext}}=J^{T}F_{\mathrm{ext}},$$ (27)

where $J\in R^{6\times n}$ is the Jacobian and $F_{\mathrm{ext}}\in R^{6}$ is the external Cartesian wrench.

### 3.6 Dynamics-compensated P–P control on the WAM teleoperation system

In a bilateral teleoperation system, the joint-space dynamics of the leader and follower robots are given in Equation 28 as

$$\begin{array}{cc} M_{l}\left(q_{l}\right){\ddot{q}}_{l}+b_{l}\left(q_{l},{\dot{q}}_{l}\right){\dot{q}}_{l}+g_{l}\left(q_{l}\right)+\tau_{d_{l}} & =\tau_{l}+\tau_{h},\\ M_{f}\left(q_{f}\right){\ddot{q}}_{f}+b_{f}\left(q_{f},{\dot{q}}_{f}\right){\dot{q}}_{f}+g_{f}\left(q_{f}\right)+\tau_{d_{f}} & =\tau_{f}-\tau_{e}, \end{array}$$ (28)

where $\tau_{l},\tau_{f}\in R^{n}$ are the control torques and $\tau_{h},\tau_{e}$ denote the human and environment interaction torques.

To implement the transparency-optimized P–P controller described in Section 3.3, the control torques are designed in Equation 29 as

$$\begin{array}{cc} \tau_{l} & =\tau_{\text{ff},l}+C_{l}\left(q_{f}-q_{l}\right),\\ \tau_{f} & =\tau_{\text{ff},f}+C_{f}\left(q_{l}-q_{f}\right), \end{array}$$ (29)

where $C_{l},C_{f}\in R^{n\times n}$ are diagonal position-feedback gains and the feedforward terms compensate the intrinsic robot dynamics. Using the inverse-dynamics model described in Section 3.4, the controller is implemented as Equation 30

$$\begin{array}{cc} \tau_{l} & =Y_{l}\left(q_{f},{\dot{q}}_{f},{\ddot{q}}_{f}\right){\hat{\pi }}_{b_{l}}+C_{l}\left(q_{f}-q_{l}\right),\\ \tau_{f} & =Y_{f}\left(q_{l},{\dot{q}}_{l},{\ddot{q}}_{l}\right){\hat{\pi }}_{b_{f}}+C_{f}\left(q_{l}-q_{f}\right), \end{array}$$ (30)

where ${\hat{\pi }}_{b_{l}},{\hat{\pi }}_{b_{f}}$ are the estimated dynamic parameter vectors. Equivalently, the feedforward compensation can be written in inverse-dynamics form as given in Equation 31

$$\begin{array}{cc} \tau_{l} & ={\hat{M}}_{l}\left(q_{l}\right){\ddot{q}}_{f}+{\hat{b}}_{l}\left(q_{l},{\dot{q}}_{l}\right){\dot{q}}_{f}+{\hat{g}}_{l}\left(q_{l}\right)+{\hat{\tau }}_{d_{l}}+C_{l}\left(q_{f}-q_{l}\right),\\ \tau_{f} & ={\hat{M}}_{f}\left(q_{f}\right){\ddot{q}}_{l}+{\hat{b}}_{f}\left(q_{f},{\dot{q}}_{f}\right){\dot{q}}_{l}+{\hat{g}}_{f}\left(q_{f}\right)+{\hat{\tau }}_{d_{f}}+C_{f}\left(q_{l}-q_{f}\right), \end{array}$$ (31)

where ${\hat{M}}_{i}\left(q\right)$ , ${\hat{b}}_{i}\left(q,\dot{q}\right)$ , ${\hat{g}}_{i}\left(q\right)$ , and ${\hat{\tau }}_{d_{i}}$ denote the estimated inertia, Coriolis/centrifugal, gravity, and disturbance/friction terms of robot i∈{l,f} , respectively. For analysis, the feedback terms are written in PD form as given in Equation 32

$$\begin{array}{cc} C_{l}\left(q_{f}-q_{l}\right) & =K_{p}^{l}\left(q_{f}-q_{l}\right)+K_{d}^{l}\left({\dot{q}}_{f}-{\dot{q}}_{l}\right),\\ C_{f}\left(q_{l}-q_{f}\right) & =K_{p}^{f}\left(q_{l}-q_{f}\right)+K_{d}^{f}\left({\dot{q}}_{l}-{\dot{q}}_{f}\right), \end{array}$$ (32)

where $K_{p}^{l},K_{p}^{f},K_{d}^{l},K_{d}^{f}$ are symmetric positive definite matrices. Define the synchronization error in Equation 33 as

$$e=q_{l}-q_{f},\;\dot{e}={\dot{q}}_{l}-{\dot{q}}_{f}.$$ (33)

Because the inverse-dynamics compensation is approximate, the mismatch between the true and estimated dynamics is collected into residual in Equation 34

$$\begin{array}{cc} r_{l} & =\left(M_{l}-{\hat{M}}_{l}\right){\ddot{q}}_{f}+\left(b_{l}-{\hat{b}}_{l}\right){\dot{q}}_{f}+\left(g_{l}-{\hat{g}}_{l}\right)+\left(\tau_{d_{l}}-{\hat{\tau }}_{d_{l}}\right),\\ r_{f} & =\left(M_{f}-{\hat{M}}_{f}\right){\ddot{q}}_{l}+\left(b_{f}-{\hat{b}}_{f}\right){\dot{q}}_{l}+\left(g_{f}-{\hat{g}}_{f}\right)+\left(\tau_{d_{f}}-{\hat{\tau }}_{d_{f}}\right), \end{array}$$ (34)

where the dependence of the dynamic terms on q and $\dot{q}$ has been omitted for notational simplicity.

Proposition 1. Assume $M_{i}\left(q_{i}\right)$ are symmetric positive definite and that ${\dot{M}}_{i}\left(q_{i}\right)-2b_{i}\left(q_{i},{\dot{q}}_{i}\right)$ are skew-symmetric. If the PD gains are symmetric positive definite and the residual terms satisfy Equation 35

$$\|r_{l}\|\leq {\bar{r}}_{l},\;\|r_{f}\|\leq {\bar{r}}_{f},$$ (35)

then the synchronization error dynamics are uniformly ultimately bounded.

Proof. Substituting the control law into the robot dynamics yields Equation 36

$$\begin{array}{cc} M_{l}\left(q_{l}\right)\ddot{e}+b_{l}\left(q_{l},{\dot{q}}_{l}\right)\dot{e}+K_{d}^{l}\dot{e}+K_{p}^{l}e & =\tau_{h}-r_{l},\\ M_{f}\left(q_{f}\right)\ddot{e}+b_{f}\left(q_{f},{\dot{q}}_{f}\right)\dot{e}+K_{d}^{f}\dot{e}+K_{p}^{f}e & =\tau_{e}+r_{f}. \end{array}$$ (36)

Consider the Lyapunov candidate in Equation 37

$$V=\frac{1}{2}{\dot{e}}^{T}\left(M_{l}+M_{f}\right)\dot{e}+\frac{1}{2}e^{T}\left(K_{p}^{l}+K_{p}^{f}\right)e.$$ (37)

Since $M_{l}+M_{f}$ and $K_{p}^{l}+K_{p}^{f}$ are positive definite, V is positive definite. Using the skew-symmetry property gives Equation 38

$$\dot{V}=-{\dot{e}}^{T}\left(K_{d}^{l}+K_{d}^{f}\right)\dot{e}+{\dot{e}}^{T}\left(\tau_{h}+\tau_{e}\right)+{\dot{e}}^{T}\left(r_{f}-r_{l}\right).$$ (38)

Let $\lambda_{\min}\left(\cdot \right)$ denote the smallest eigenvalue of a symmetric positive definite matrix. Then Equation 39 gives

$${\dot{e}}^{T}\left(K_{d}^{l}+K_{d}^{f}\right)\dot{e}\geq \lambda_{\min}\left(K_{d}^{l}+K_{d}^{f}\right)\|\dot{e}{\|}^{2}.$$ (39)

Furthermore, Equation 40 gives

$$|{\dot{e}}^{T}\left(r_{f}-r_{l}\right)|\leq \|\dot{e}\|\left({\bar{r}}_{l}+{\bar{r}}_{f}\right).$$ (40)

Thus, Equation 41 follows

$$\dot{V}\leq -\lambda_{\min}\left(K_{d}^{l}+K_{d}^{f}\right)\|\dot{e}{\|}^{2}+\|\dot{e}\|\left(\|\tau_{h}+\tau_{e}\|+{\bar{r}}_{l}+{\bar{r}}_{f}\right).$$ (41)

Therefore, Equation 42 implies that $\dot{V}<0$ whenever

$$\|\dot{e}\|>\frac{\|\tau_{h}+\tau_{e}\|+{\bar{r}}_{l}+{\bar{r}}_{f}}{\lambda_{\min}\left(K_{d}^{l}+K_{d}^{f}\right)}.$$ (42)

Consequently, Equation 43 gives

$${\mathrm{lim sup}}_{t\to \infty }\|\dot{e}\left(t\right)\|\leq \frac{\|\tau_{h}+\tau_{e}\|+{\bar{r}}_{l}+{\bar{r}}_{f}}{\lambda_{\min}\left(K_{d}^{l}+K_{d}^{f}\right)},$$ (43)

which establishes uniform ultimate boundedness of the synchronization error.

In the ideal case, Equation 44 requires

$$r_{l}=r_{f}=0,\;\tau_{h}+\tau_{e}=0,$$ (44)

and Equation 45 becomes

$$\dot{V}=-{\dot{e}}^{T}\left(K_{d}^{l}+K_{d}^{f}\right)\dot{e}\leq 0.$$ (45)

The closed-loop error dynamics reduce to a damped second-order system, and Equation 46 implies

$$e\left(t\right)\to 0,\;\dot{e}\left(t\right)\to 0.$$ (46)

Thus, the leader and follower robots asymptotically synchronize in the absence of modeling errors and interaction disturbances. 

The proposed control law relies on reasonably accurate inverse-dynamics models of the robots. In practice, modeling inaccuracies, unmodeled friction effects, and acceleration-estimation errors appear as bounded residual disturbances $r_{l}$ and $r_{f}$ in the synchronization error dynamics. Under this assumption, Proposition 1 establishes uniform ultimate boundedness of the synchronization error, while exact inverse-dynamics compensation yields asymptotic synchronization. Communication and hardware-induced delays are assumed negligible, consistent with the experimental setup considered in this work.

**FIGURE 3:**
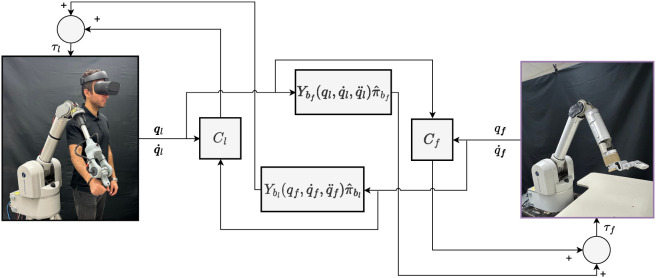
Schematic of the transparency-optimized position–position control architecture on the WAM bilateral teleoperation system. Figure contains images of the author(s) only.

Figure 3 illustrates a schematic of the proposed dynamics-compensated P–P controller implemented on the WAM bilateral teleoperation system. Details regarding gain tuning for passivity are provided in Section 4.3.

## 4 Experiments

### 4.1 Experiment setup

The bilateral teleoperation system consists of a 7-degree-of-freedom (DOF) Barrett WAM arm acting as the follower and a 4-DOF Barrett WAM arm equipped with a 3-DOF custom-designed haptic wrist on the leader side (Figure 1). The haptic wrist is designed to mirror the kinematics of the WAM’s wrist, providing intuitive orientation feedback to the operator. The cable-driven mechanism of WAM arms gives them high back-drivability and low friction compared to gear-driven robotic arms, such as KUKA LBR or Universal Robots (UR) series. In addition, the WAM platform features a relatively low moving mass for a human-scale robotic arm, which improves responsiveness and reduces operator effort. These mechanical properties make the WAM particularly well suited for transparency-focused teleoperation experiments. Nevertheless, the intrinsic dynamics of the WAM remain perceptible during free motion and interaction, motivating the use of explicit dynamic compensation as described in Section 3.

The control software was implemented in C++ using the open-source libbarrett library, which provides low-level access to the WAM hardware, including joint position, velocity, and torque interfaces. Custom teleoperation control loops were implemented on top of this framework to realize both the baseline and the proposed transparency-optimized P–P controllers. Communication between the leader and follower robots was established through UDP sockets on the same computer, minimizing network-induced delays. With this setup, the controller operates at a sampling frequency of 500 Hz, which is sufficient to ensure stable bilateral control. The implementation of the teleoperation controllers is publicly available at https://github.com/amir-noohian/wam-teleop/tree/2c-dyntorq.

### 4.2 Inverse dynamics estimation

In this section, the base dynamic parameters of the leader and follower WAM robots are identified. Because the effects of wrist dynamics are comparatively small, they are neglected in the modeling. The wrist joints are therefore treated as single rigid links, and only the first four DOFs are considered for parameter estimation in both robots. The parameters of the leader and follower are estimated independently using the open-source implementation provided in Sousa and Cortesão (2013).

**FIGURE 4:**
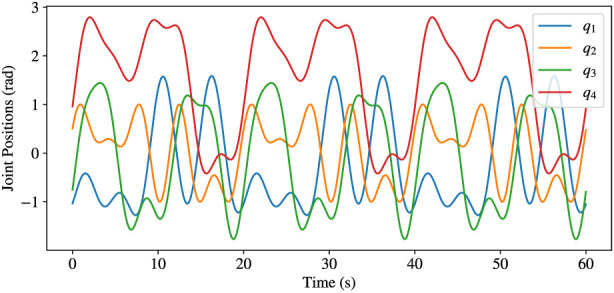
Optimized excitation trajectories for the four joints.

For estimation, each WAM robot is excited using the optimal excitation trajectory described in Section 3.4. The trajectory of each joint is represented by a truncated Fourier series with M=5 harmonics and fundamental angular frequency $\omega_{f}=0.1\pi$ rad/s. The amplitudes of the Fourier coefficients are optimized to minimize the condition number of the regressor matrix while satisfying the joint limits of the robot. The resulting excitation trajectories for the four joints are shown in Figure 4. The trajectory is executed at a sampling frequency of 500 Hz for a duration of 60 s, resulting in approximately 30,000 data samples for each identification experiment. During the excitation motion, joint positions, velocities, and torques are recorded, while joint accelerations are obtained from the velocities using a central difference scheme. To reduce measurement noise, a low-pass filter with a cutoff frequency of 2.5 Hz is applied to the measured signals before parameter identification. Using the recorded joint states and torques, the base parameters are estimated following the method described in Section 3.4. According to the modeling approach, a 4-DOF WAM robot has 26 identifiable base parameters.

To validate the identified parameters, five random reference trajectories were executed on each robot, and the joint states were recorded. The accuracy of the inverse dynamic model was evaluated by computing the Normalized Root Mean Square Error (NRMSE) between the measured torques $\tau_{\mathrm{ref}}$ and the torques predicted by the estimated model, $\tau_{\mathrm{est}}=Y_{b}\left(q_{\mathrm{ref}},{\dot{q}}_{\mathrm{ref}},{\ddot{q}}_{\mathrm{ref}}\right){\hat{\pi }}_{b}$ . Validation was performed separately for the leader and follower, and the results are summarized in Table 1. The low NRMSE values confirm the reliability of the estimated parameters and demonstrate that the identified models accurately capture the dynamic behavior of both robots.

**TABLE 1:** NRMSE (mean (std), %) between the measured torques and the estimated torques from the base parameters.

Joint i	Leader WAM arm	Follower WAM arm
Joint 1	5.25 (0.34)	5.34 (1.07)
Joint 2	2.31 (0.20)	2.79 (0.55)
Joint 3	5.69 (2.50)	5.46 (0.72)
Joint 4	3.69 (0.39)	3.01 (0.34)

### 4.3 Experimental modeling and gain tuning for passivity

Beyond transparency, stability and passivity are essential requirements for safe bilateral teleoperation. A passive teleoperation system does not inject energy into the human–robot–environment loop, thereby preventing unintended oscillations that may endanger the operator, the robotic hardware, or the environment. In this work, the controller parameters in (Equation 30) were tuned experimentally to ensure passive behavior of the proposed dynamic-compensated P–P control scheme. Communication delays were not considered in this study; instead, the focus was placed on stability and passivity issues arising from modeling assumptions and controller implementation.

The tuning process was guided by two key experimental observations. First, incorporating stiction and Coulomb friction terms into the inverse dynamics model was found to inject energy into the teleoperation system, resulting in loss of passivity. Despite testing several friction modeling approaches, consistently passive behavior could not be achieved when these terms were included. As a result, only viscous friction was retained in the dynamic model, as discussed in Section 3.4.

Second, directly using estimated joint accelerations in the feedforward dynamics compensation caused instability, which was attributed to noise in the acceleration estimates. To mitigate this effect, the acceleration term was scaled prior to being used in the inverse dynamics model. Empirically, using 25% of the estimated joint accelerations consistently resulted in stable and passive behavior while preserving the benefits of dynamic compensation.

Although the acceleration term in the inverse dynamics model is scaled and explicit stiction and Coulomb friction terms are omitted, the resulting controller remains well suited for practical human-scale teleoperation tasks. Typical human-operated teleoperation involves relatively slow, smooth motions rather than aggressive accelerations, making the reduced contribution of high-frequency inertial effects acceptable in practice. Likewise, many everyday teleoperation tasks do not require extremely delicate or fine manipulation dominated by frictional interactions. As a result, the proposed modeling and control choices provide a favorable balance between stability, passivity, and transparency, while remaining effective for a wide range of realistic teleoperation scenarios.

Table 2 summarizes the position control gains used in the experiments. Identical gains were applied to both the leader and follower WAM robots. Only the first four degrees of freedom were actively controlled, while the wrist joints were locked and modeled as a single rigid link. These gains were used consistently across all experiments reported in this paper.

**TABLE 2:** Position control gains for the first four joints of the WAM robots.

Joint i	1	2	3	4
$k_{p,i}$	750	1000	400	200
$k_{d,i}$	8.3	8	3.3	0.8

### 4.4 Evaluation procedure of control architectures

We follow the experimental transparency evaluation framework of Aliaga et al. (2004), described in Section 3.1, to assess key teleoperation performance parameters. The evaluation is conducted in joint space, consistent with the controller implementation, and all experiments are performed without a human operator to ensure repeatability and objectivity.

#### 4.4.1 Free motion

Known feedforward joint torques are applied to the leader robot, and the resulting joint positions are recorded. Tracking performance is evaluated using the normalized root mean square error (NRMSE) between the leader and follower joint positions, which should ideally approach zero. The leader impedance is computed as the ratio ${\hat{\tau }}_{h,RMS}/\delta q_{l,RMS}$ , where ${\hat{\tau }}_{h}$ denotes the estimated external joint torque at the leader obtained using the sensorless external torque estimation method described in Section 3.5, $\delta q_{l}$ represents the change in the leader joint position, and RMS denotes the root mean square. Ideally, the leader impedance in free motion should approach zero. Two torque magnitudes are applied three times each, resulting in six trials.

#### 4.4.2 Hard contact

**FIGURE 5:**
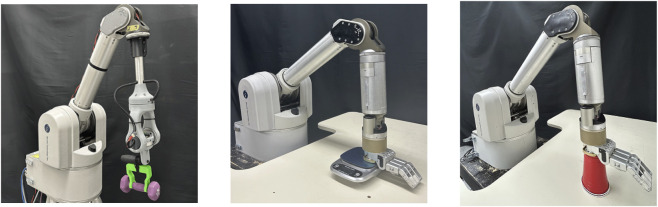
Evaluation experimental setup: weights mounted on the leader’s end-effector (left), the follower in contact with the kitchen scale (middle), and the follower interacting with a plastic cup in the compression task (right).

Force tracking and maximum transmittable impedance are evaluated during contact with the environment. Calibrated weights are mounted on the leader end-effector (Figure 5 (left)), while the follower end-effector is pressed against a kitchen scale with 0.1 g resolution (Figure 5 (middle)). The robots are configured such that the last link is perpendicular to the ground, ensuring normal contact. The mounted weights induce a contact force that is transmitted to the follower side. Force tracking is quantified using the NRMSE between the estimated external joint torques of the leader and follower, denoted by ${\hat{\tau }}_{h}$ and ${\hat{\tau }}_{e}$ , respectively, which should ideally approach zero. The maximum transmittable impedance is evaluated as ${\hat{\tau }}_{h,RMS}/RMSE\left(q_{l},q_{f}\right)$ , where RMSE denotes the root mean square error. In hard contact, this impedance should ideally approach infinity. Two weight magnitudes are tested three times each, resulting in six trials.

#### 4.4.3 External torque estimation

Ground-truth interaction forces are obtained from the mounted weights on the leader side and the scale measurements on the follower side. Using (Equation 27), Cartesian forces are mapped to joint torques through the Jacobian transpose. Estimation accuracy is quantified using the NRMSE between the estimated and ground-truth joint torques.

Due to the kinematic structure of the WAM arms, the second and fourth joints contribute most significantly to the workspace and are particularly sensitive to dynamic effects. These joints also play a dominant role during contact interactions. Therefore, for clarity of presentation, only results corresponding to joints 2 and 4 of both the leader and follower robots are reported.

### 4.5 Comparison of different controllers

The objective evaluation described in Section 4.4 is used to compare the performance of the proposed Feedforward Dynamics Compensation + PD (DC) controller with the baseline Gravity Compensation + PD (GC) controller, which corresponds to the default control mode of the WAM arms. In addition to the baseline architecture, we further compare DC with transparency-enhancement methods used in human-scale teleoperation: GC-FF and GC-LFB, which apply force feedforward (Fahmi and Hulin, 2018) and local force feedback (Hastrudi-Zaad and Salcudean, 1999; Balachandran et al., 2020) on the leader, respectively, on top of GC. These methods are implemented in the joint space using the estimated external torque. A gain of 0.75 is used for both, as higher gains caused instability. To ensure a meaningful comparison, all controllers include gravity compensation, since operating a human-scale robotic arm using a pure PD position controller would be impractical and would not yield informative results.

Table 3 compares the performance of the controllers in free motion. Each experimental condition was repeated n=6 times, and results are reported as mean ± standard deviation. Statistical significance between controllers was evaluated using a two-sided Welch t -test with significance level α=0.05 . The proposed DC controller significantly outperforms the GC controller in both joint position tracking accuracy and leader-side impedance (p<0.05) . Specifically, the joint position tracking error is reduced by approximately 32% for joint 2 and 57% for joint 4, while the leader impedance decreases by about 66% for joint 2 and 56% for joint 4. The observed differences correspond to large effect sizes (Cohen’s d>0.8 ). The GC-FF and GC-LFB controllers provide partial improvements over GC, particularly by reducing the leader-side impedance. However, their impact on position tracking accuracy is limited and less consistent, and overall they still fall short of the performance achieved by the DC controller. These results indicate that incorporating full dynamic compensation is necessary to substantially improve transparency, resulting in smoother operation and reduced resistance during unconstrained motion.

**TABLE 3:** Free-motion performance metrics: position tracking error and leader’s impedance (mean (std)).

Controller	Position tracking error (NRMSE, %)		Leader’s impedance (N ⋅ m/rad)	
	Joint 2	Joint 4	Joint 2	Joint 4
GC	0.688 (0.069)	0.920 (0.122)	31.738 (6.294)	3.060 (0.422)
GC-FF	0.713 (0.064)	0.870 (0.113)	18.302 (0.859)	1.908 (0.092)
GC-LFB	0.587 (0.087)	1.090 (0.373)	18.742 (3.904)	3.039 (0.959)
DC	0.470 (0.053)	0.393 (0.038)	10.831 (1.783)	1.351 (0.148)

Table 4 compares the controllers under hard-contact conditions. Each experimental condition was repeated n=6 times, and the reported values correspond to mean ± standard deviation. Statistical significance between controllers was evaluated using a two-sided Welch t -test with significance level α=0.05 . Both the GC and DC controllers exhibit comparable maximum transmittable impedance, indicating that the perceived stiffness during rigid contact is largely unchanged. This behavior is expected, as hard contact involves minimal motion and the performance is fundamentally limited by the position–position teleoperation structure. Force tracking performance, however, shows a moderate improvement with the DC controller, with statistically significant differences (p<0.05) . Specifically, the force tracking error is reduced by approximately 53% for joint 2 and 56% for joint 4. The observed differences correspond to large effect sizes (Cohen’s d>0.8 ). In contrast, the GC-FF controller degrades performance in hard contact, resulting in higher force tracking errors and a noticeable reduction in maximum transmittable impedance compared to GC. The GC-LFB controller, on the other hand, exhibits performance comparable to GC in both force tracking accuracy and maximum transmittable impedance.

**TABLE 4:** Hard-contact performance metrics: force tracking error and maximum transmittable impedance (mean (std)).

Controller	Force tracking error (NRMSE, %)		Max. Transmittable impedance (N ⋅ m/rad)	
	Joint 2	Joint 4	Joint 2	Joint 4
GC	11.350 (4.401)	9.573 (4.809)	1026.464 (64.222)	216.831 (29.336)
GC-FF	84.990 (5.040)	92.340 (4.749)	579.428 (16.745)	117.448 (7.449)
GC-LFB	7.643 (3.770)	14.467 (9.869)	1029.322 (25.936)	205.135 (17.226)
DC	5.380 (2.237)	4.233 (1.941)	1038.238 (10.010)	219.590 (3.291)

Overall, the experimental results demonstrate that feedforward dynamics compensation significantly enhances free-motion transparency by reducing both position tracking error and leader-side impedance, while preserving stable and consistent behavior during hard contact. This confirms the theoretical findings that dynamic compensation effectively addresses the transparency limitations of the basic P–P architecture in free motion, while the remaining limitations in hard contact are structural rather than implementation-related.

Table 5 reports the external torque estimation error for the leader and follower. The estimation errors are small and show no significant differences across controllers, indicating that the external torque estimation method remains robust and consistent regardless of the control strategy.

**TABLE 5:** NRMSE (mean (std), %) between estimated and ground-truth external joint torques.

Controller	Leader WAM arm		Follower WAM arm	
	Joint 2	Joint 4	Joint 2	Joint 4
GC	4.19 (2.83)	3.38 (2.13)	11.14 (7.97)	10.99 (4.45)
GC-FF	6.13 (8.07)	4.83 (3.46)	1.22 (1.10)	2.62 (0.63)
GC-LFB	3.19 (3.15)	2.84 (0.11)	12.71 (7.74)	5.11 (1.81)
DC	5.44 (1.27)	3.77 (2.83)	5.95 (12.69)	9.54 (3.79)

Finally, it should be emphasized that the notion of perfect transparency must be interpreted carefully in practical systems. The theoretical analysis in Section 3 considers two limiting cases: a basic PID position controller and an idealized controller with perfect feedback linearization. In practice, dynamic compensation lies between these extremes. Gravity compensation removes constant bias forces, and the proposed DC controller further compensates inertial and Coriolis effects using estimated dynamics. While modeling uncertainties prevent achieving perfect transparency, the proposed approach represents a practical and effective step toward it.

### 4.6 Case study: compression task

In this section, we evaluate the proposed feedforward dynamics compensation (DC) controller on a teleoperation task that consists of two distinct phases: free motion and interaction with a remote environment. A human operator uses the leader device to guide the follower robot through free space toward an object and subsequently bring it into contact with it. Specifically, the follower interacts with and compresses a plastic cup (Figure 5 (right)), forming a contact-rich scenario that requires precise control of the remote environment. This task structure allows evaluation of teleoperation performance in both unconstrained motion and contact conditions. The operator performs the task continuously, transitioning from free motion to contact within a single trial, enabling a qualitative assessment of how the teleoperation system behaves across the two operating regimes. The proposed DC controller is compared with three teleoperation systems introduced in Section 4.5.

**FIGURE 6:**
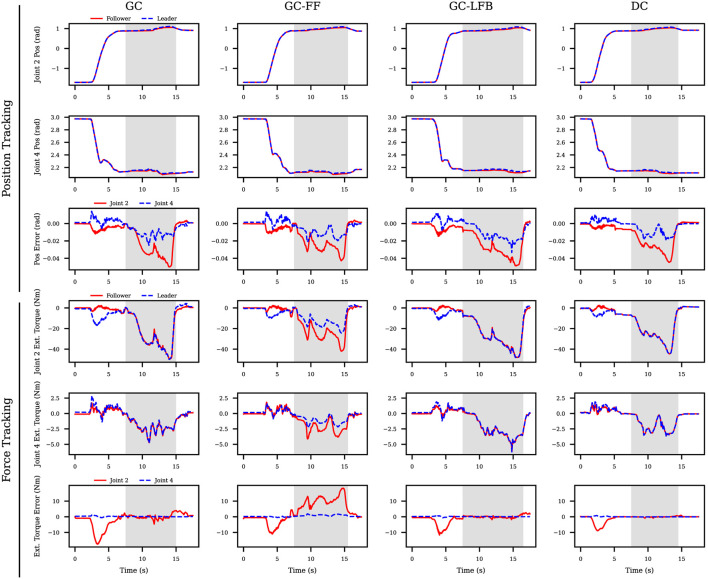
Comparison of teleoperation systems using joint positions for position tracking and external joint torques for force tracking. Gray regions indicate the contact period.

Figure 6 shows position and force tracking during the task, with the gray area indicating the contact phase. During free motion, the DC controller exhibits smoother motion and improved force tracking compared to GC, reflecting a lower perceived leader impedance and reduced resistance to operator input. The GC-FF and GC-LFB controllers provide partial improvements over GC during this phase, but their behavior remains less consistent than that of DC. During contact, all controllers maintain stable interaction with the environment. However, GC-FF exhibits larger force tracking errors, indicating degraded performance. GC-LFB shows behavior largely comparable to GC, whereas DC provides more consistent position and force responses. These observations qualitatively support the quantitative results presented in the previous section.

Overall, this case study demonstrates that the proposed DC controller improves free-motion transparency while maintaining stable behavior during contact, supporting its applicability to practical teleoperation tasks involving transitions between unconstrained motion and contact with the environments.

## 5 Conclusion

This paper presented a transparency-optimized position–position (P–P) control architecture for bilateral teleoperation systems. By leveraging estimated inverse dynamics as a feedforward compensation term, the proposed approach improves transparency without introducing additional sensors or hardware, while preserving the inherent simplicity and stability of the P–P scheme.

Experimental validation on a human-scale WAM bilateral teleoperation system demonstrated that the proposed controller significantly enhances free-motion transparency. Compared to a gravity-compensated P–P controller, joint position tracking error was reduced by up to 32% and leader-side impedance by up to 56%, resulting in a smoother and more natural operating experience. In hard-contact scenarios, the proposed method maintained comparable maximum transmittable impedance while achieving up to 53% improvement in force tracking accuracy. These results confirm that dynamics-based compensation can effectively improve practical transparency in P–P teleoperation systems, particularly for human-scale manipulators where inertia and damping strongly influence operator perception.

Future work will focus on improving the dynamic model, including more accurate friction representation, and incorporating online adaptation or disturbance observers to enhance robustness against modeling errors and external disturbances. Another important direction is to evaluate the proposed controller under communication delays and packet loss, and to investigate delay-robust or passivity-based compensation strategies to ensure stability and transparency in realistic teleoperation scenarios.

## Data Availability

The raw data supporting the conclusions of this article will be made available by the authors, without undue reservation.
